# Physically and Chemically Cross-Linked Poly(vinyl alcohol)/Humic Acid Hydrogels for Agricultural Applications

**DOI:** 10.1021/acsomega.3c05868

**Published:** 2023-11-15

**Authors:** Ana V. Torres-Figueroa, Sergio de los Santos-Villalobos, Dora E. Rodríguez-Félix, Sergio F. Moreno-Salazar, Cinthia J. Pérez-Martínez, Lerma H. Chan-Chan, Andrés Ochoa-Meza, Teresa del Castillo-Castro

**Affiliations:** † Departamento de Investigación en Polímeros y Materiales, Universidad de Sonora, Hermosillo 83000, Mexico; ‡ Laboratorio de Biotecnología del Recurso Microbiano, Departamento de Ciencias Agronómicas y Veterinarias, Instituto Tecnológico de Sonora, 5 de Febrero 818 Sur, Colonia Centro, Obregón 85000, Mexico; § Departamento de Agricultura y Ganadería, Universidad de Sonora, Carr. Bahía de Kino, Km. 21. Apartado Postal 305, Hermosillo, Sonora 83000, Mexico; ∥ Departamento de Ciencias Químico Biológicas, Universidad de Sonora, Hermosillo 83000, Mexico; ⊥ Departamento de Física, CONAHCyT, Universidad de Sonora, Hermosillo 83000, Mexico

## Abstract

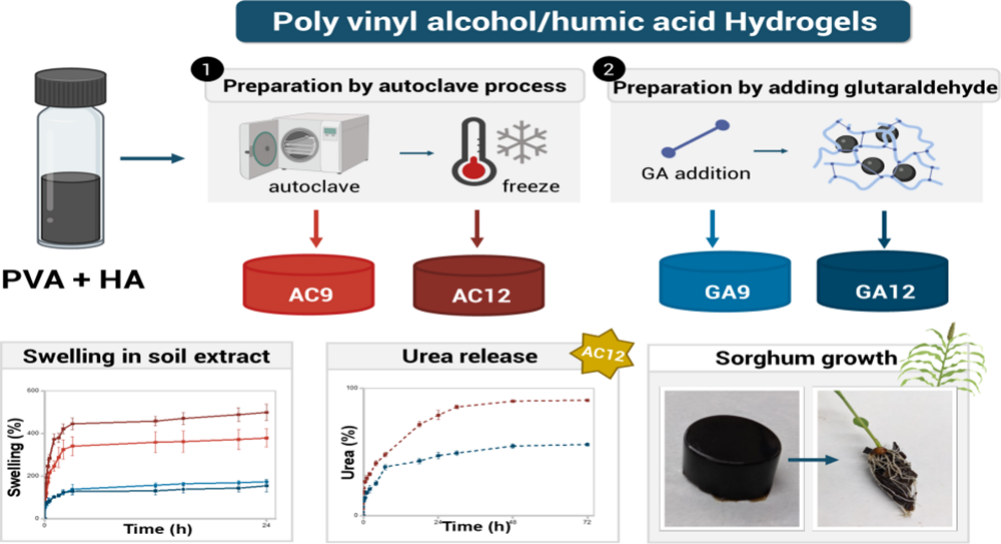

The preparation method of hydrogels has a significant effect on their structural and physicochemical properties. In this report, physically and chemically cross-linked poly(vinyl alcohol) (PVA) networks containing humic acid (HA) were alternatively prepared by autoclaving (AC) and through glutaraldehyde (GA) addition, respectively, for agricultural purposes. PVA/HA hydrogels were comparatively characterized by Fourier transform infrared spectroscopy, thermogravimetric analysis, mechanical assays, scanning electron microscopy, swelling kinetics measurements, and water retention tests in soil. AC hydrogels showed a more homogeneous porous microstructure, higher swelling levels, and a better capacity to preserve the humidity of soil than those obtained by adding GA. Both PVA/HA hydrogels exhibited no phytotoxicity on cultivation trials of *Sorghum sp.*, but the plant growth was promoted with the GA-cross-linked network as compared to the effect of the AC sample. The release behavior of urea was modified according to the preparation method of the PVA/HA hydrogels. After 3 days of sustained urea release, 91% of the fertilizer was delivered from the AC hydrogel, whereas a lower amount of 56% was released for the GA-cross-linked hydrogel. Beyond the advantages of applying PVA/HA hydrogels in the agricultural field, an appropriate method of preparing these materials endows them with specific properties according to the requirements of the target crop.

## Introduction

Hydrogels are three-dimensional (3D) hydrophilic polymer networks, which can be formed by physical or chemical cross-linking methods.^1^ These materials are capable of absorbing and retaining large amounts of water without being solubilized, as well as their polymer networks can expand or contract according to their environmental conditions.^2^ Hydrogels have been extensively applied in controlled drug delivery,^3^ tissue engineering,^4^ biosensors,^5^ and agriculture,^6^ among other fields. The physicochemical properties of hydrogels depend on the inherent characteristics of polymer matrixes, proportion of monomers or polymers, their cross-linking nature, and the experimental setup for their preparation.^7^

In the agricultural area, hydrogels have been intended to retain water in crops, for moisture conservation, to mitigate plant drought stress, in seed coating, and for the controlled release of fertilizers. The porous structure of these materials promotes the availability of oxygen and water in the root system, stimulating the physiological parameters of the plant and promoting its growth.^8−10^

Poly(vinyl alcohol) (PVA) is a candidate for plant growth substrate uses.^11^ PVA-based hydrogels have been widely developed owing to the feasible tune of their preparation methods, low toxicity, high water absorption, and mechanical stability.^7^ Typically, PVA hydrogels have been prepared by physical cross-linking, through a series of freeze–thaw cycles and anneal-swell, or by adding chemical cross-linking agents, such as bifunctional aldehydes.^7,12−14^

The preparation method modulates the physicochemical properties of PVA hydrogels and their behavior in the agricultural applications.^15,16^ For example, Sarkar and Sen synthesized physically cross-linked PVA hydrogels with poly(ethylene glycol) and sodium sulfate in the presence of urea.^17^ The hydrogel showed a sustained release of urea and a high absorption of Fe^3+^ ions from soil. Hakim et al.^18^ prepared chemically cross-linked PVA hydrogels containing a nanoclay filler and loading with a potassium phosphate fertilizer. They reported that the addition of the nanoclay improved the dispersion of potassium phosphate in the PVA matrix, controlling the rate and amount of fertilizer release.

Humic acid (HA) macromolecules have been used in agricultural fields as the nutrient and promoter of plant growth.^19^ This material is formed by macromolecules derived from humic substances, which are organic matter distributed in the soil, natural water, and sediments, resulting from the decomposition of plants and natural residues.^20^ Due to their amphiphilic character, HA molecules form micelle-like structures under neutral to acidic conditions, which are useful in agriculture,^21^ pollution remediation,^22^ medicine,^23^ and the pharmaceutical industry.^24^ HA molecules have undefined compositions that vary according to their origin and production process. The typical composition of HA includes high proportions of ionizable phenol and carboxyl groups, containing also quinones as well as sugar and peptide residues.^20^ Some reports have mentioned that the addition of HA to hydrogels can beneficially modulate their mechanical behavior.^25,26^

Combining PVA frameworks with HA molecules seems to be a convenient alternative to form a multifunctional material for agricultural purposes. However, few studies have dealt with this approach. A previous report showed the synthesis of PVA-based supramolecular hydrogels containing HA nanoparticles extracted from leonardite using a cyclic freeze–thaw method. In this study, it was shown that HA nanoparticles physically interact with PVA chains and interconnect polymer chains as a functional cross-linker.^13^

Considering the potential of the PVA/HA combination as precursors of biomaterials with advantages for the agricultural area, this work presents the preparation of HA-containing PVA hydrogels by alternative processes of autoclaving (AC) and glutaraldehyde (GA) addition. The hydrogels were comparatively characterized by Fourier transform infrared spectroscopy (FTIR), thermogravimetric analysis (TGA), compression test, scanning electron microscopy (SEM), swelling kinetic measurements, and water retention evaluation in soil. The effect of the different PVA/HA hydrogels on the growth and physiological traits of sorghum plants was also evaluated. Finally, the release kinetics of urea from the hydrogels were evaluated in a soil aqueous extract. Urea is a common fertilizer that is typically lost due to volatilization and leaching, increasing the eutrophication of aqueous environments.^27^ The absence of phytotoxicity of PVA/HA hydrogels and their sustained urea release profiles evidenced the potential of the materials for agricultural applications.

## Materials and Methods

### Materials

PVA (Mw 85,000–124,000, 99% hydrolyzed); HA sodium salt, technical grade; acetic acid, 99.7%; urea, 98%, and GA solution grade II, 25% in H_2_O, were purchased from Sigma-Aldrich. All reagents were of analytical grade and used as received without further purification. The aqueous solutions were prepared with deionized water and purified by a Milli-Q Organex system (Millipore, Molsheim, France).

### Preparation of Hydrogels by the Autoclave Process

A PVA solution (5 wt %) was prepared by dissolving PVA in deionized water for 3 h at 85 °C. An amount of HA powder (9 or 12 wt % for AC9 or AC12, respectively) was dispersed in 2 mL of the PVA solution. The pH of the mixture solution was adjusted to 4.0 using acetic acid. Then, the solution was poured into cylindrical molds and placed in a vertical autoclave (CVQ-B50L) at 120 °C and 0.12 MPa for 90 min.^28^ Subsequently, the solution was allowed to cool at room temperature and placed at −12 °C for 12 h. Finally, hydrogels AC9 and AC12 were removed from the mold, washed with deionized water, and dried by lyophilization in a Labconco FreeZone freeze-dryer of 4.5 L.

### Preparation of Hydrogels by Adding Glutaraldehyde

To prepare the GA cross-linked hydrogels, an amount of HA powder (9 or 12 wt % for GA9 or GA12, respectively) was dispersed in 2 mL of a 5 wt % PVA solution. The pH of the mixture solution was adjusted to 4.0 using acetic acid, followed by the addition of 50 μL of GA with further stirring. The resultant suspension was poured into cylindrical molds and allowed to cure at room temperature for 24 h.^14^ The hydrogels GA9 and GA12 were removed from molds, washed with deionized water, and dried by lyophilization.

**Figure 1:**
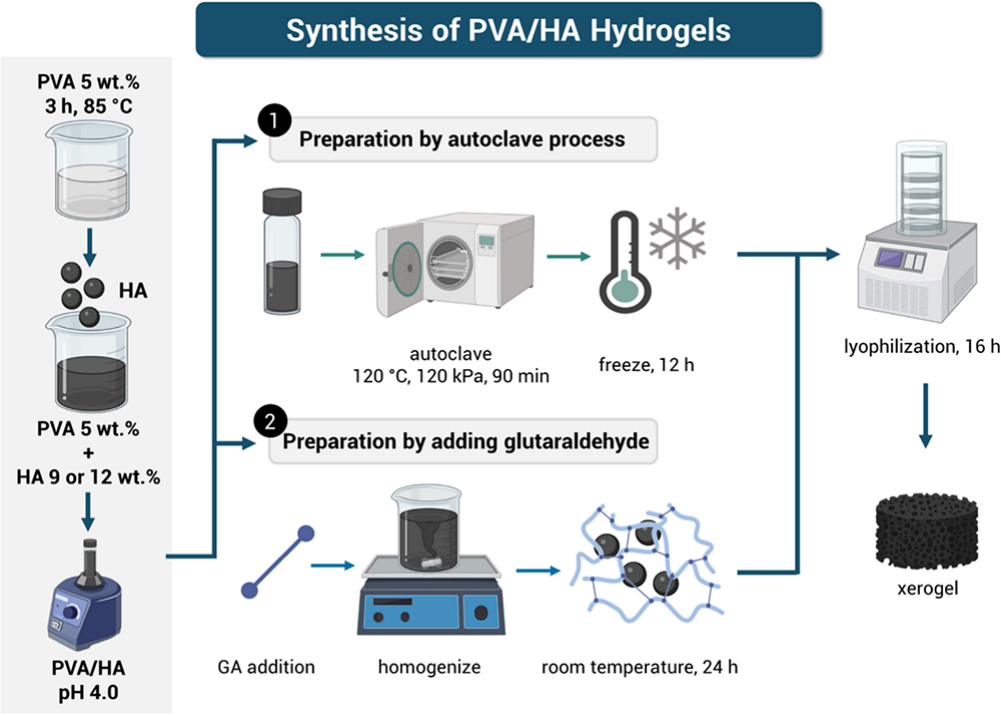
PVA/HA hydrogel preparation methods.

Table 1 summarizes the component portions used for the preparation of each hydrogel type. The hydrogel code numeral indicates the HA wt % in dried samples. Figure 1 illustrates the preparation methods for PVA/HA hydrogels.

**Table 1:** Feed Compositions in the Preparation of Hydrogels

sample	PVA 5 wt % (mL)	HA (mg)	GA (μL)
AC9	2	9.89	
AC12	2	13.63	
GA9	2	9.89	50
GA12	2	13.63	50

### Hydrogel Characterization

FTIR spectra were recorded in a Frontier spectrometer (PerkinElmer, Beaconsfield, UK) by the KBr pellet technique in the range of 4000–500 cm^–1^. The spectra of commercial reagents were also included to identify their inherent peaks in the hydrogel spectra.

TGA experiments were carried out under a nitrogen flow until 800 °C and a heating rate of 10 °C min^–1^ using a Pyris 1 apparatus (PerkinElmer, Llantrisant, UK).

Mechanical properties of hydrated hydrogels were evaluated in compression tests using a TA ElectroForce 5500 BioDynamic equipment with a 200 N load cell. Cylindrical-shaped hydrogels of 10 mm diameter × 7 mm height were cyclic-loaded 5 times up to 50% deformation at a constant strain rate of 3.5 mm s^–1^.^29^ GA and AC hydrogels without adding HA were prepared and labeled as GA0 and AC0, respectively.

SEM was used to study the internal morphology of the hydrogel samples. The analyses were performed using a scanning electron microscope model JEOL JSM-5410LV (JEOL-LTD, Tokyo, Japan) operated with an acceleration voltage of 15 kV. Cross-sectional samples were frozen and lyophilized. Then, the dried samples were fixed on carbon ribbon and gold sputtered prior to SEM examination.

### Swollen Polymer Network Modeling Calculations

Thermodynamic equations were used to estimate structural parameters of the GA hydrogels. A density kit was 3D-printed using poly(lactic acid) on a Dremel 3D40 printer (3PI Tech Solutions, Illinois, USA).^30^ Polymer volume fractions in swollen (ϕ*s*) and relaxed (ϕ*r*) states were calculated by the following equations:
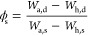

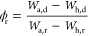
where *W*_a,d_ is the hydrogel weight in the dry state in air, *W*_h,d_ is the weight in the dry state in heptane, *W*_a,s_ is the weight in the swollen state in air, *W*_h,s_ is the weight in the swollen state in heptane, *W*_a,r_ is the weight in the relaxed state in air, and *W*_h,r_ is the weight in the relaxed state in heptane.

The effective molecular weight between cross-links (*M̅*_c_) was determined by the Bray–Peppas–Merrill modified equation of Flory–Rehner as follows^31,32^:
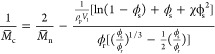


The frequency of chain-end defects (γ) was also calculated according to the following equation^30^:
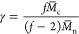


The shear modulus (*G*) was calculated according to the rubberlike elasticity theory using the following equation^30,33^:
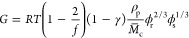


The hydrogel mesh size (ξ), which defines the linear distance between consecutive cross-links, was calculated using a modified Canal–Peppas equation^34^:
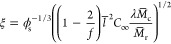


The parameters used for modeling calculations are provided in Table S1.

### Swelling Properties

The swelling capacity of hydrogels was evaluated in deionized water and in a soil aqueous extract (pH 7.05, EC = 239 μS cm^–1^) by the gravimetric method. The soil extract was prepared according to Durpekova et al.^35^ Commercial garden soil (Happy Flower-prepared potting soil mix) was autoclaved at 120 °C and 0.12 MPa for 40 min. Then, 20 g of soil was added to 1 L of deionized water, and the resultant suspension was stirred for 24 h. Afterward, the suspension was centrifuged at 3540 rpm for 10 min. The supernatant was used in the swelling experiments.

Freeze-dried samples of known weight (*W*_0_) were immersed in aqueous media. At specific times (*t*), the samples were removed from the swelling medium, blotted, weighed (*W*_*t*_), and placed in the same bath until a constant weight was reached. The swelling percent at time *t* was calculated from the following relation:
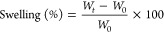


Data of swelling kinetics were fitted to the Korsmeyer–Peppas model to elucidate the mechanism of swelling in the hydrogels:
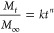


In this equation, *M*_*t*_/*M*_*∞*_ represents the fractional uptake of solvent normalized with respect to the equilibrium conditions. The variables *k* and *n* are constants which can be related to diffusion coefficients and the specific transport mechanism.^36^

### Water Retention Capacity

The ability of hydrogels to retain water in soil was assessed by measuring the water evaporation ratio (WER). Commercial garden soil was dried at 60 °C for 48 h in a mechanical convection laboratory incubator (Thermo Scientific Precision 3511, USA). The dried hydrogel samples were buried in 20 g (*W*_0_) of the dried soil in a plastic pot. Pure soil was used as control. Then, 20 mL of deionized water was added to each pot, and their weights (*W*_1_) were monitored. The samples were stored at room temperature and their weights monitored at different times (*W*_*t*_) until no detectable weight loss was observed.^35,37^ The WER percent at time *t* was calculated from the following relation:
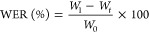


### Effect of Hydrogels on Plant Growth

A phytotoxicity test was carried out to evaluate the effect of the hydrogel on growth and physiological traits of plants. The test was performed according to the methodology reported by Montesano et al.^6^ Seeds of sorghum (*Sorghum sp.*) were placed in conical centrifuge tubes with common tissue paper or with hydrogel samples. The paper was moistened with deionized water (control), whereas hydrogel samples were saturated with deionized water. The tubes were maintained at 27 ± 1 °C and a photoperiod of 12 h light using a plant growth chamber (Percival Scientific model CU36L4, Iowa, USA). Plant growth was observed after 3, 5, and 7 days. An amount of 30 replications (tubes) was performed per each treatment.

After the growth observation time, the seedlings were cut crosswise, separating the aerial part from the roots. Then, both parts were dried in a mechanical convection laboratory incubator (Thermo Scientific Precision 3511, USA) for 48 h at 60 °C. Once dry, samples were weighed, and the average dry weight of the aerial part and the roots is herein reported. For comparative purposes, analysis of variance (ANOVA) was carried out with an acceptable level of significance of *P* < 0.05 using the statistical package Minitab19.

### Loading and Controlled Release of the Fertilizer from the Hydrogel

The loading of urea in the hydrogels was carried out by the swelling equilibrium method. Freeze-dried samples were loaded by sorption in 0.5 mL of an aqueous solution of urea (0.1 M) for 48 h followed by lyophilization.^37^ The urea concentration was quantified with an enzymatic kit (Randox Laboratories Limited, Crumlin, UK), measuring the absorbance at 685 nm in an UV–Vis spectrophotometer, model 8453 (Agilent Technologies, Shangai, China).

The urea release kinetics were obtained in the soil aqueous extract (pH 7.05, EC 239 μS cm^–1^). Urea-loaded hydrogels were immersed in 30 mL of the release medium at a controlled temperature of 25 °C using a shaking water bath model LSB-030s (LabTech, Gyeonggi-do, Korea) with continuous orbital stirring of 60 rpm. Aliquots of 20 μL were withdrawn at specific time intervals and replaced with equal volumes of fresh medium.^38,39^ The urea concentration was quantified as previously mentioned.

### Mathematical Modeling of Urea Release

Data obtained in the urea release studies were fitted to the Korsmeyer–Peppas model, as previously specified, to evaluate the mechanism of release of the chemical compound from the hydrogels. In this case, *M*_*t*_ and *M*_*∞*_ were the absolute cumulative amounts of urea released at time *t* and equilibrium, respectively; *k* was the release rate coefficient, and *n* was the diffusional exponent that can be related to the chemical compound transport mechanism.^14,40^

## Results and Discussion

### FTIR Spectroscopy

**Figure 2:**
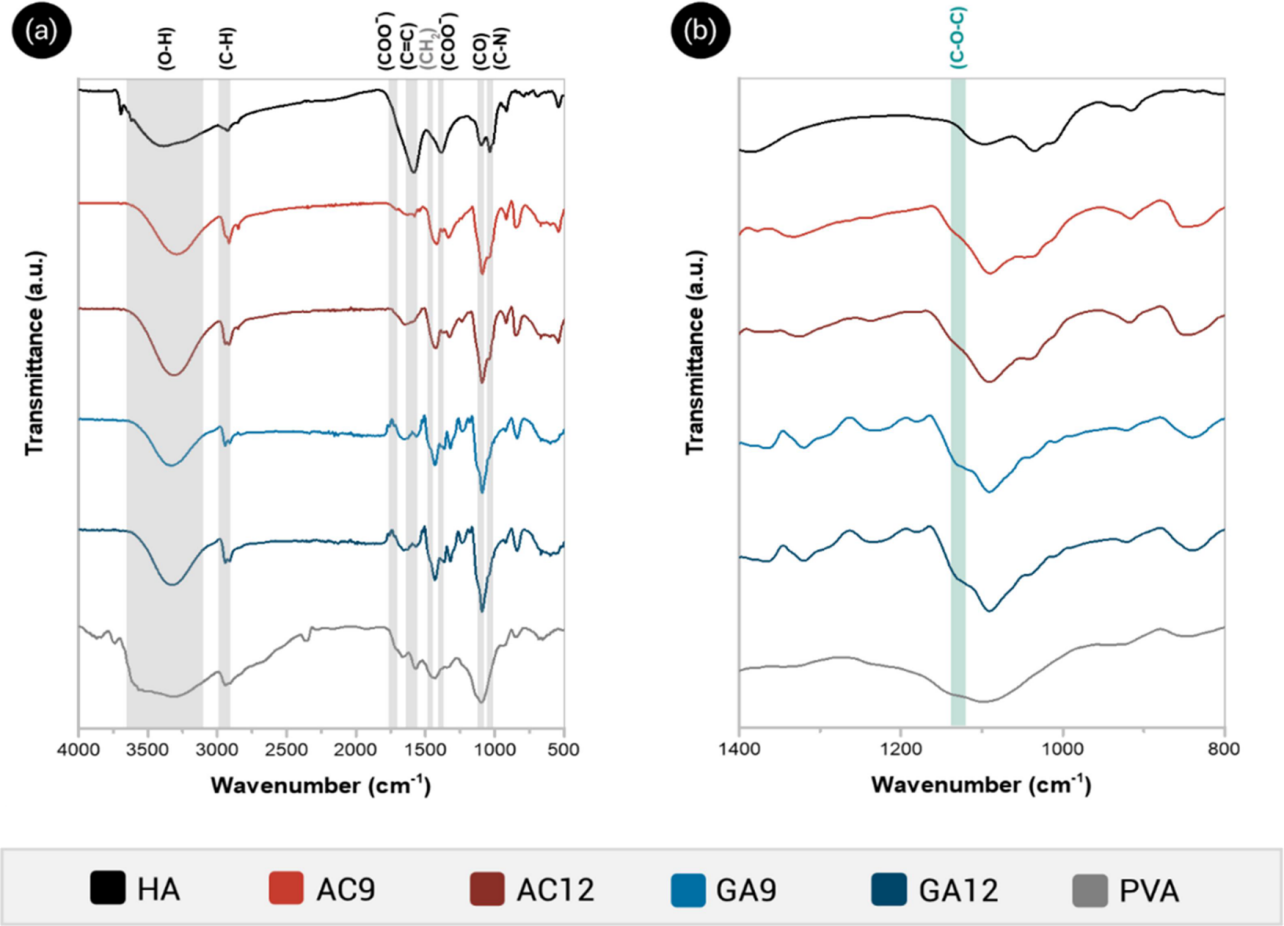
FTIR spectra of PVA, HA, and composite hydrogels; full spectra from 4000 to 500 cm^–1^ (a) and spectral details from 1400 to 800 cm^–1^ (b).

Figure 2a shows the FTIR spectra of the PVA/HA hydrogels and those of their neat PVA and HA components. Table 2 summarizes the assignments of FTIR bands of PVA and HA samples.

**Table 2:** Assignment of the FTIR Bands^a^

sample	wavenumber (cm^–1^)	functional groups
PVA	3700–3180	ν (O–H)
	2941	ν (C–H)
	1430	δ (CH_2_)
	1099	ν (C–O)
HA	3385	ν (O–H)
	2927	ν (C–H)
	1690	ν (COO^–^)
	1585	ν (C=C)
	1394	ν (COO^–^)
	1106	ν (CO)
	1035	ν (C–N)

a Abbreviations: ν, stretching; δ, bending.

PVA spectrum shows the typical broadband attributed to the stretching vibration of the O–H bond in the 3700–3180 cm^–1^ region. The signals corresponding to the stretching vibration of the C–H bond are located around 2941 cm^–1^. The peak related to the wagging of CH_2_ group appears at 1430 cm^–1^, while the peak at 1099 cm^–1^ is attributed to the stretching vibration of the C–OH moiety.^14^

The HA spectrum shows a broadband at 3385 cm^–1^ attributed to the O–H vibrational stretching of the hydrogen-bonded carboxyl, alcohol, and phenol groups. The band at 2927 cm^–1^ is attributed to the asymmetric C–H stretching vibration of the methyl and/or methylene groups. A small shoulder at 1690 cm^–1^ is related to the COO^–^ stretching vibration corresponding to carboxyl groups, and a band at 1585 cm^–1^ is due to the C=C vibrational stretching mode. The signals at 1394, 1106, and 1035 cm^–1^ are attributed to COO^–^ moieties, C–O stretching vibration of phenolic groups, and C–N stretching vibration, respectively.^13^

Most of the absorptions of PVA and HA overlap in the spectra of composite hydrogels; however, some individual signals are also distinguished. It is noticed that the peak intensity ratio of O–H and C–O signals (I_O–H_/I_C–O_) was higher in PVA/HA spectra, with values of 1.011, 1.029, 1.015, and 1.017 for AC9, AC12, GA9, and GA12, respectively, than for the PVA sample (1.000).

In the case of hydrogels prepared by AC, individual peaks of HA moieties are observed, particularly those signals attributed to C=C (1572 cm^–1^) and COO– (1332–1326 cm^–1^) stretching vibrations. Furthermore, this band of carboxyl groups appeared at lower wavenumbers in AC hydrogel spectra with respect to that of the HA sample (1394 cm^–1^) indicating physical interactions between components, for example, hydrogen bonding between carboxyl groups of the HA and the hydroxyl side groups of the PVA chains.^13^ During the AC stage, the thermal and pressure conditions promoted the intermolecular contact between PVA and HA. Next, a self-supporting hydrogel was built by phase separation of the PVA/HA mixture under water-freezing conditions, leading to a polymer-rich phase in which the close interchain contact promoted intermolecular hydrogel bonding and crystallite formation. The crystalline regions remained intact after the sample was thawed at room temperature and a three-dimensional supramolecular network of PVA was formed in which the HA molecules were trapped by additional physical interactions between the active sites of both components.^13,41^

**Figure 3:**
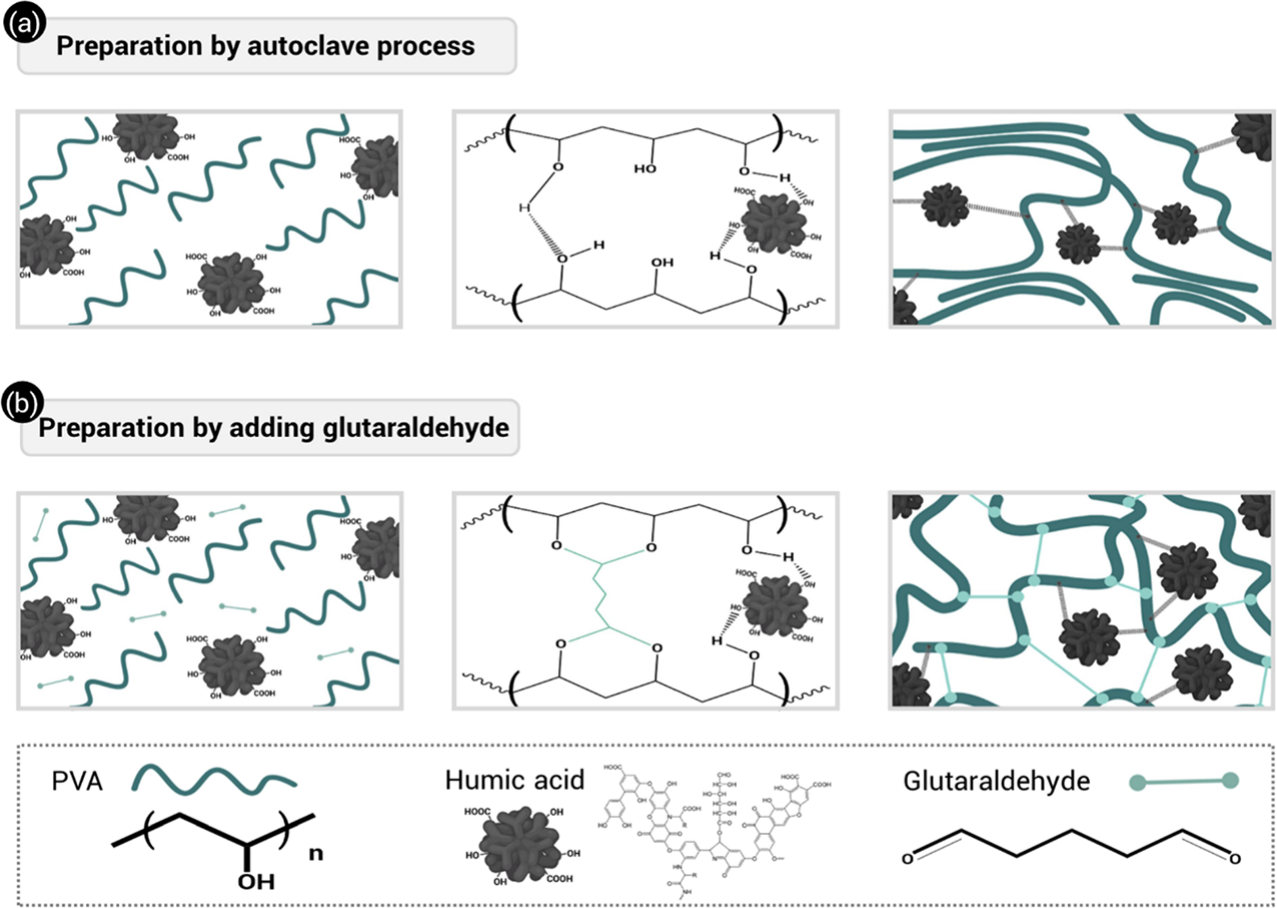
Schematic representation of AC PVA/HA hydrogel (a) and GA PVA/HA hydrogel (b) formation.

In the case of spectra of hydrogels formed by adding GA, the peak at 1120 cm^–1^ is attributed to the symmetric C–O–C stretching of the acetal ring resulting from the cross-linking reaction between PVA and GA^14^ (Figure 2b). Figure 3 illustrates the interactions between the components of the hydrogels prepared by both methods.

### TG Analysis

**Figure 4:**
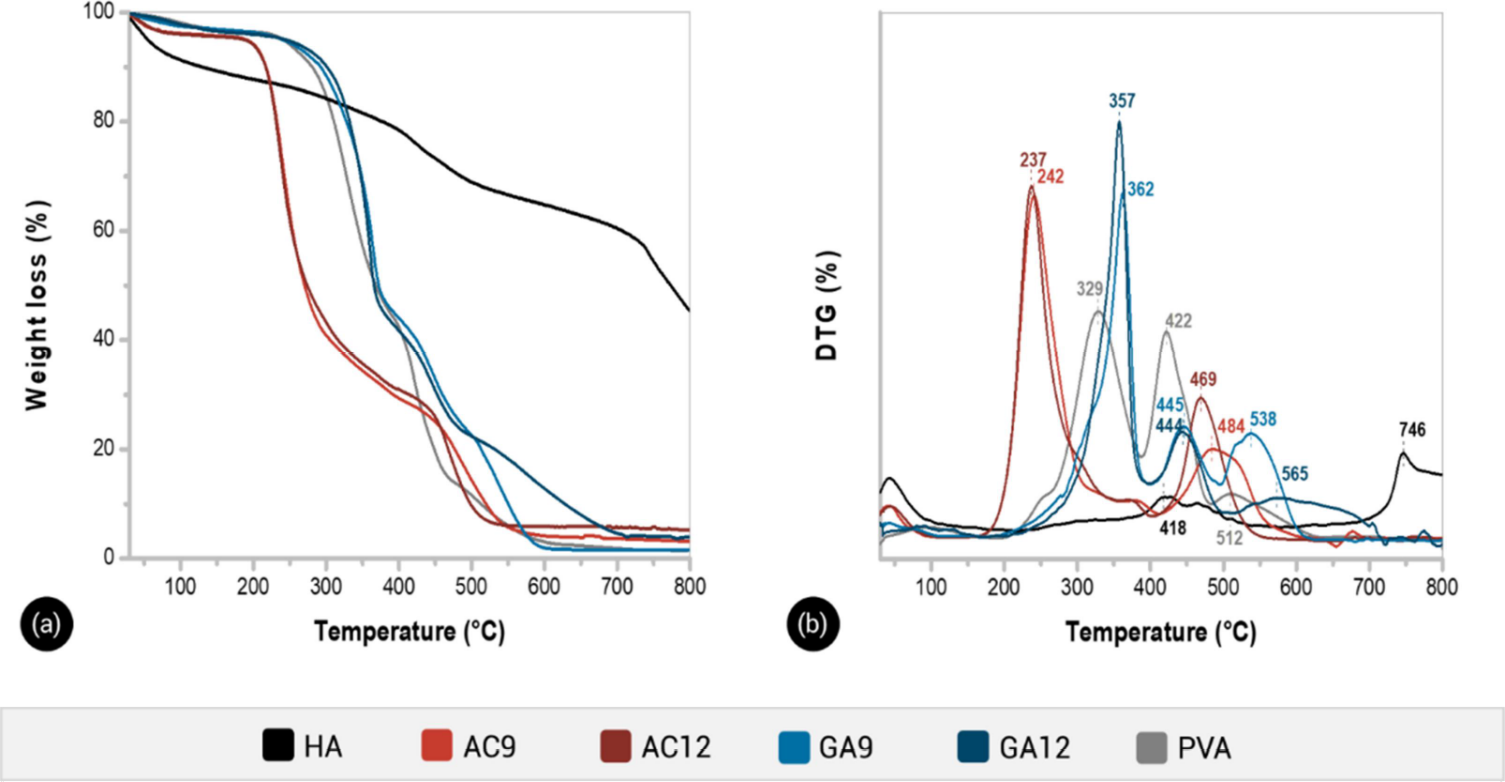
Thermogravimetric (a) and first derivative curves (b) for hydrogel composites and their individual components.

Figure 4a shows the thermograms of PVA, HA, and hydrogel samples of different compositions. Figure 4b shows the temperatures of the maximum rate of weight loss (*Tmax*) for each weight loss step for different materials.

All samples lost mass at temperatures below 100 °C, which was associated with the evaporation of residual moisture. PVA exhibited a three-step degradation process (*Tmax* of 328.6, 421.9, and 512.0 °C) as previously reported.^42^ The first stage of degradation was related to the elimination reactions that occur in linear and aliphatic polymers, yielding polyene compounds through dehydration, including the degradation of the hydroxyl side groups. Since the degradation temperature of the first stage was not high enough to break all main-chain bonds, polyene intermediates were degraded into low-molecular-weight products in the next degradation step. The second thermal degradation step involved a complex set of reactions that include chain cleavage, side, and cyclization reactions as well as the continuous degradation of residual acetate groups due to the breakdown of the polymer backbone. The last stage of degradation was related to the decomposition of carbon and hydrocarbons residues formed during the second stage.^43^

The HA exhibited a multistep degradation process, showing a higher thermal stability than the PVA at high temperatures (i.e., more than 50% of HA mass is preserved after heating up to 800 °C). Different decomposition reactions can occur during HA heating according to its heterogeneous chemical composition. Thermal-induced reactions involved the decomposition of carboxyl, methyl, methylene, and alcohol groups, as well as the decomposition of carbohydrate units and oxidation/polycondensation of aromatic structures.^44^

Composite hydrogels showed degradation temperatures similar to those of their counterparts. The *T*_max_ of the first step of weight loss of AC formed hydrogels shifted more than 100 °C to lower temperatures as compared to the neat PVA sample. The low thermal stability of AC hydrogels was straightly associated with the decrease of the molecular weight of PVA due to the AC conditions (120 °C, 120 kPa).^45^

Conversely, the main weight loss step of GA9 and GA12 samples occurred at *T*_max_ of 357 and 362 °C, respectively, that was at least 28 °C higher than the *T*_max_ of neat PVA (329 °C). The shift of the degradation peaks to higher temperatures for samples GA9 and GA12 indicated that the chemical cross-linking effectively improved the thermal stability of the hydrogels as compared to linear PVA and physically cross-linked samples. The acetal bridge between PVA chains increased the thermal requirements for scission reactions.

### Mechanical Behavior of Hydrogels

All hydrogels exhibited J-shaped stress–strain behavior with high compliance at low strains and high strength at high strains. This behavior can be suitable for the mechanical adaptation of materials to the action of external forces, like those occurring in soil. At 50% deformation, none of the hydrogels showed fracture, and all samples recovered their original shape once the applied load was released (Figure S1).

**Figure 5:**
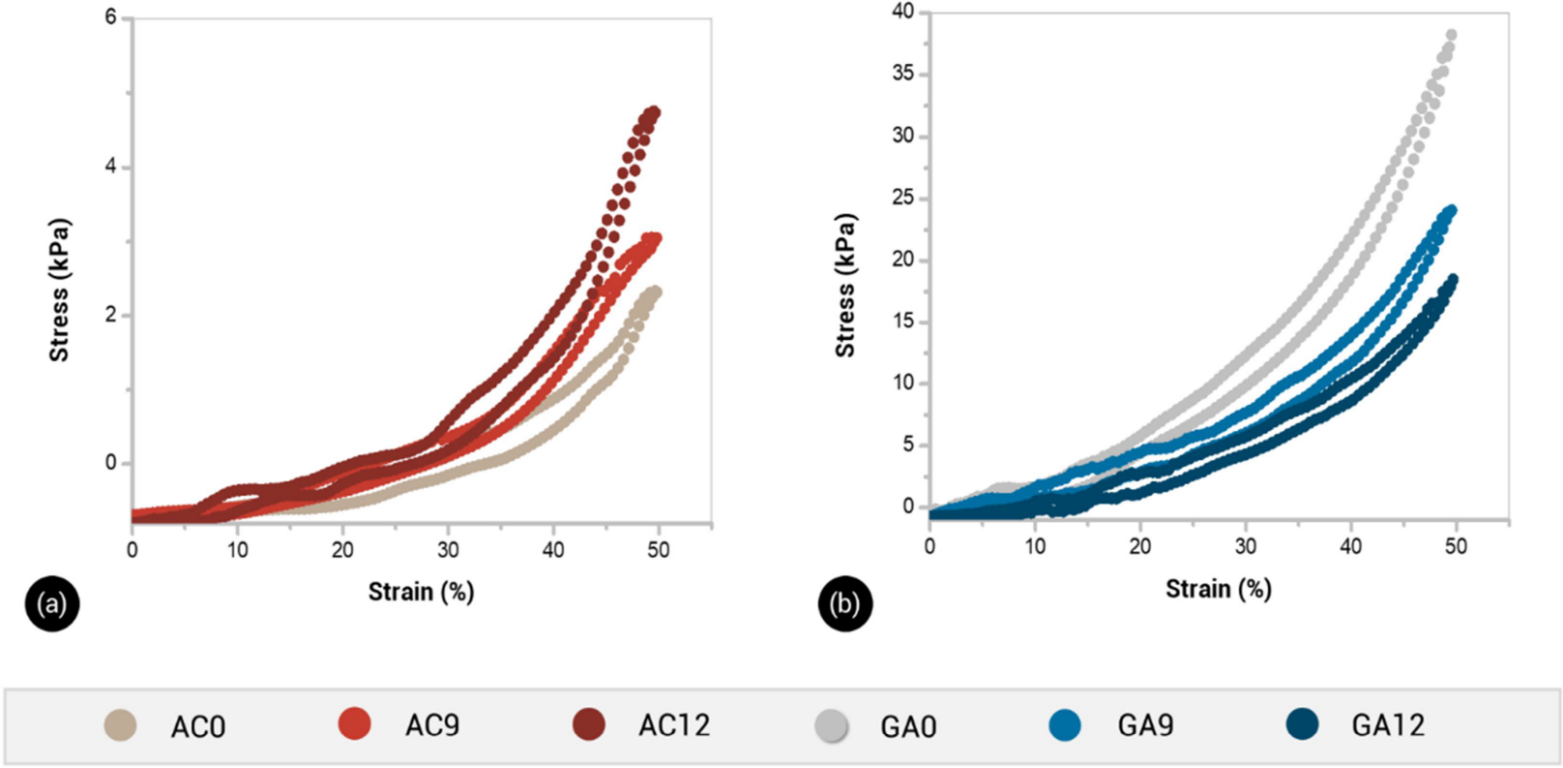
Representative compressive stress–strain curves for AC (a) and GA hydrogels (b).

The compressive stress–strain curves are displayed in Figure 5. For AC hydrogels, the compressive strength at 50% strain increased with an increase in the HA content in the hydrogel (Figure 5a). This results evidenced that HA molecules strengthened the supramolecular network of PVA by additional interactions with PVA moieties, increasing the interconnections of PVA chains.^13^

GA hydrogels exhibited higher strength values than the AC hydrogels in the whole strain range (Figure 5b). Moreover, the compressive strength of GA samples at 50% strain decreased with an increase in the HA content in hydrogel samples. It should be noticed that the maximum compressive strength of the GA12 hydrogel was 52% lower than that of the GA0 sample. It is suggested that the presence of HA molecules during the chemical formation of the 3D-network of PVA partially hindered the reaction between the GA cross-linker and the hydroxyl side groups of PVA chains.

### SEM Analysis

**Figure 6:**
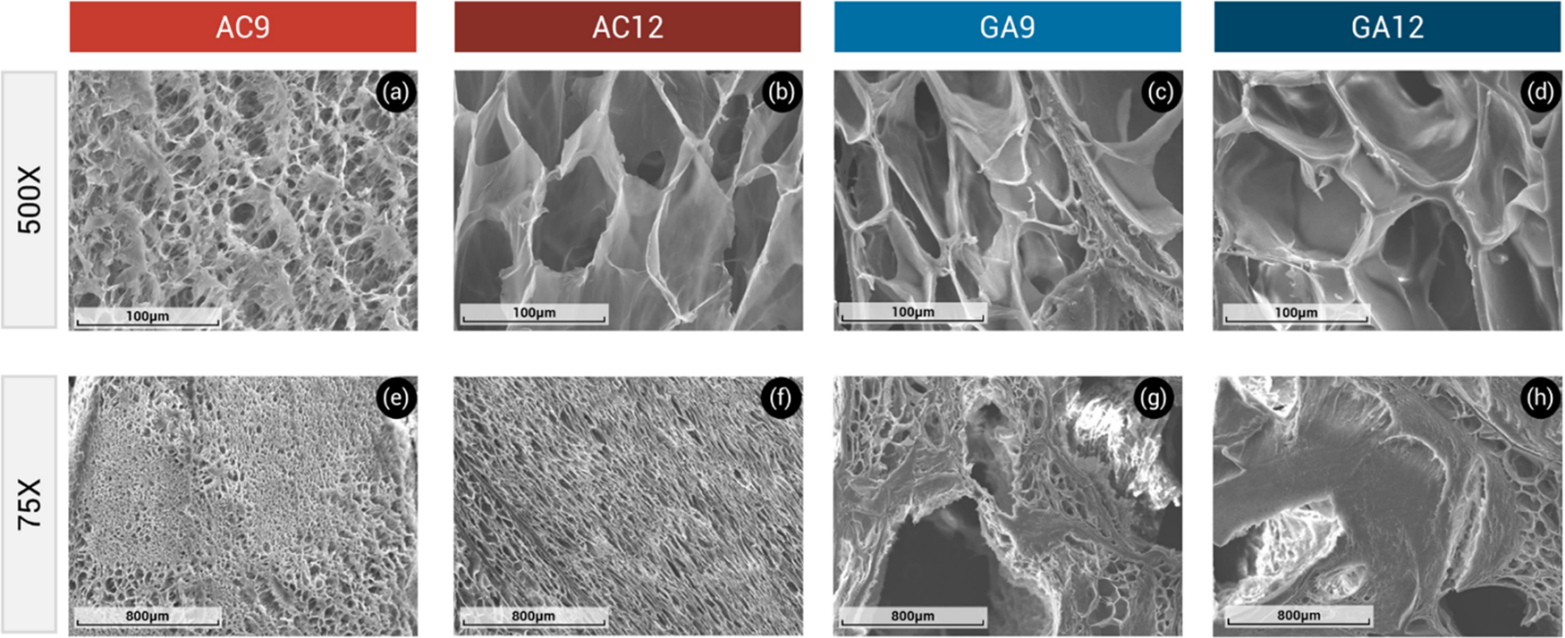
SEM micrographs of cross sections of AC9 (a,e), AC12 (b,f), GA9 (c,g), and GA12 (d,h) hydrogels at 500X (a–d) and 75X (e–h) magnifications.

Figure 6 shows SEM micrographs of cross sections of freeze-dried PVA/HA hydrogels at different magnifications. A porous structure in the form of a well-defined network is observed in all samples. This interconnected porous structure has been observed in chemically and physically cross-linked PVA hydrogels.^13,14,16,46^

For autoclaved hydrogels, the increase of HA content increased the pore dimensions (Figure 6a,b,e,f), indicating a reorganization of PVA phase by adding HA. During freezing, ice crystals were formed, leading to PVA and HA-rich domains.^7,46^ These results differ from those obtained by Sirousazar and Khodamoradi who found that by increasing the HA ratio in freeze-thawed PVA/HA hydrogels, their pore size was reduced.^13^Figure S2a,b shows the pore size distribution of AC9 and AC12 hydrogels, respectively.

The pore dimensions of GA-cross-linked PVA/HA hydrogels were larger than those of their analogous autoclaved hydrogels (Figure S2c,d). This effect was more noticeable in hydrogels containing 9 wt % HA (Figure 6c,d,g,h). Furthermore, GA-cross-linked PVA/HA hydrogels exhibited regions of different porous sizes, evidencing a less homogeneous porous microstructure as compared to autoclaved PVA/HA hydrogels. The nonuniform structures of chemical PVA hydrogels have been associated with the rapid kinetics of cross-linking reactions and the inhomogeneous dispersion of the cross-linker.^7^ The SEM results suggested that the preparation method of the PVA/HA hydrogels played a significant role in controlling the size and shape of their porous structure.

### Evaluation of Hydrogel Structural Parameters

The poor thermal stability of the CA samples showed that the autoclave conditions strongly affected the molecular weight of the PVA chains; thus, the polymer network modeling calculations were carried out only for GA hydrogels.

Table 3 summarizes the results obtained from the swollen polymer network modeling calculations. The values of *M̅*_*c*_ and mesh size indicated that the GA9 sample exhibited a lower distance between network junctions compared to that of the GA0 and GA12 samples. The mesh size of the GA12 sample (5.06 nm) was higher than that calculated for the GA9 network (3.98 nm); however, without adding HA, the mesh size of the PVA network was the highest (5.67 nm). The higher values of mesh sizes in the GA0 and GA12 samples compared to that of GA9 hydrogels may favor the loading of high molecular weight compounds within their network structures.^47^

**Table 3:** GA Hydrogel Formulation Structural Parameters at 25 °C

sample	ϕ_s_	ϕ_r_	*M̅*_c_ (g mol^–1^)	γ	*G* (kPa)	ξ (nm)
**GA0**	0.092	0.058	1463.81	0.028	70.70	5.67
**GA9**	0.067	0.027	585.71	0.011	97.15	3.98
**GA12**	0.071	0.032	982.15	0.018	65.69	5.06

The theoretical shear modulus of GA hydrogels was determined to compare the relative behavior of samples with the experimental results obtained in compression mode. The theoretical shear modulus increased for the samples in the order: GA12, GA0, and GA9. Both theoretical and experimental results agreed that the GA12 hydrogel is the sample with the lowest stiffness. However, no correlation between the theoretical and experimental results was found for the GA0 and GA9 samples. The differences between theorical estimations and experimental results may be attributed to a nonideal cross-linking behavior.^47^

### Swelling Measurements

**Figure 7:**
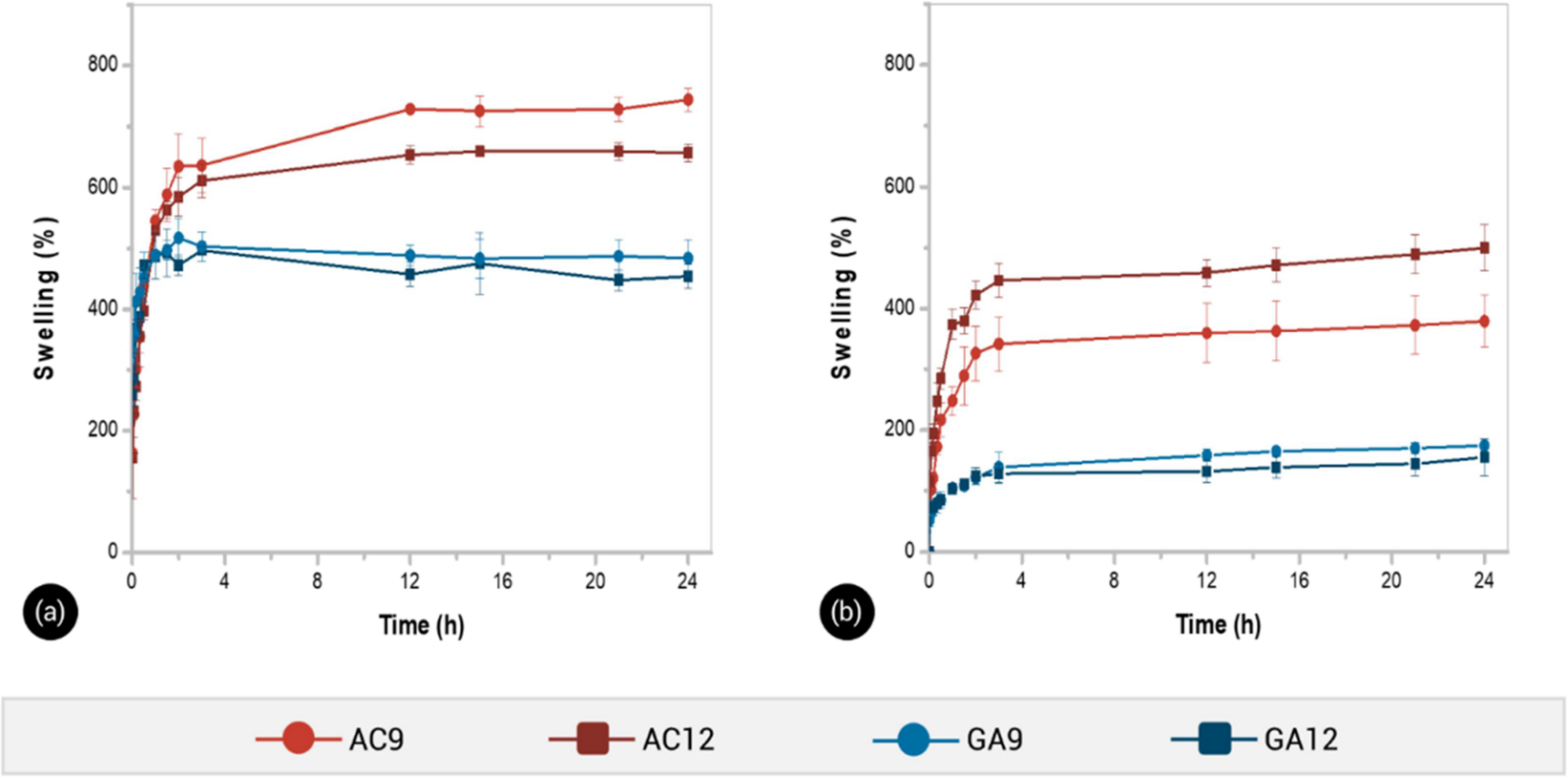
Swelling kinetics of composite hydrogels in deionized water (a) and soil extract (b) at 25 °C.

The swelling profiles were obtained for each hydrogel at room temperature in deionized water and a soil extract. Figure 7 shows the swelling kinetics of the PVA/HA hydrogels in both media. A rapid swelling occurred for all samples within the first hour due to the surface hydrophilicity and capillarity of pores of the hydrogels. Both samples AC9 and AC12 reached the swelling equilibrium at 12 and 15 h in deionized water and soil extract, respectively. The swelling equilibrium of hydrogels GA9 and GA12 was reached more faster (3 h in deionized water and soil extract) than in the case of autoclaved hydrogels. Several studies have explained that hydrogel water uptake depends on various prevailing conditions such as cross-linking density, ionic strength, temperature, and preparation method, among others.^15,48,49^

Autoclaved hydrogels exhibited a higher swelling capacity in both media than those obtained by GA cross-linking. In deionized water, hydrogels AC9 and AC12 reached swelling levels of 546 and 530%, respectively, during the first hour, while samples GA9 and GA12 reached both around 489% (Figure 7a). Furthermore, the equilibrium swelling values were 744 and 657% for AC9 and AC12, respectively, whereas the values decreased up to 484 and 454% for GA9 and GA12, respectively. This comparative swelling behavior was consistent with the SEM observations. The pore density of lyophilized AC samples was higher than that of GA hydrogels; thereby, a higher inner network space allowed the containing of more water molecules. The comparative swelling results between AC and GA samples were also consistent with their experimental mechanical behavior, evidencing that the cross-linking degree of GA hydrogels was higher than that for AC hydrogels. Increasing the cross-linking density decreased the ability of the polymer network to swell, which in turn disfavored the water uptake.^13^

Similar findings for the swelling behavior of hydrogels were observed in the soil extract (Figure 7b), although the AC12 sample reached the highest value of swelling at 24 h (500%) followed by AC9, GA9, and GA12 hydrogels with 379, 174, and 155%, respectively. The swelling level of all samples was slightly reduced as compared to those measured in deionized water. At high ionic strength, the concentration of ions increases the osmotic pressure of the hydrogel, causing water to desorb from the network.^48^ This behavior (Gibbs Donnan effect) is a consequence of a charge imbalance between the inner and outer environments of the hydrogel.^50^ In the soil extract, the concentration gradient of ion species between the hydrogel and the external solution was lower than in water medium; thus, the water uptake decreased.^35^

To further examine the swelling mechanism of the hydrogels, experimental data were fitted to the power law model. For cylinder-shaped gels, when the *n* value is less than 0.45, the solvent uptake mechanism is governed by Fickian diffusion, and a *n* value between 0.45 and 0.89 indicates that the process follows an anomalous solvent uptake mechanism. The model is also capable of predicting Case II (*n* = 1.0) transport in anomalous diffusion and Super Case II transport (*n* > 1.0).^36^ The linear fit of the plot ln *M*_*t*_/*M*_*∞*_ against ln *t* yields the diffusion exponent (*n*), the Pearson coefficient (*r*^2^), and the diffusion constant (*k*). The results are summarized in Table 4.

**Table 4:** Fitting Parameters of Swelling Data to the Power Law Equation

hydrogel sample	conditions	fitting parameters			release mechanism
		*n*	*k*	*r*^2^	
AC9	deionized water	0.3617	1.826	0.9671	Fickian diffusion
	soil extract	0.4279	2.0548	0.9716	Fickian diffusion
AC12	deionized water	0.3579	1.7825	0.995	Fickian diffusion
	soil extract	0.3057	1.6176	0.9915	Fickian diffusion
GA9	deionized water	0.1869	0.8421	0.8627	Fickian diffusion
	soil extract	0.1886	1.326	0.9693	Fickian diffusion
GA12	deionized water	0.1061	0.6086	0.8936	Fickian diffusion
	soil extract	0.1966	1.2369	0.9523	Fickian diffusion

The values of *n* indicated that the solvent sorption through the hydrogels was controlled by a Fickian diffusion mechanism in all samples under both conditions. It was also observed that the *k* values in deionized water were higher for AC samples than for the GA hydrogels, indicating a faster diffusion of water in the AC hydrogels. Furthermore, the *k* values in soil extract were higher than those obtained in deionized water, excepting for the AC12 hydrogel, in which the *k* value in soil extract was 10.2% lower than in deionized water. This behavior evidenced that the conditions of the swelling medium can modulate the diffusion of solvents in PVA/HA hydrogels.

### Water Retention Study in Soil

The water evaporation from soil is mediated by ambient conditions such as air temperature, relative humidity, and the capacity of the soil to retain water.^35^ The water retention capacity of soils produces positive effects on the survival rate of seedlings and the growth of plants.^51,52^ Hydrogels hinder the exchange of water vapor between the porous soil and atmosphere, decreasing the evaporation rate. The presence of hydrogels also helps to preserve the moisture content of agricultural soils after irrigation or rain since water is gradually released over time.^53−55^

**Figure 8:**
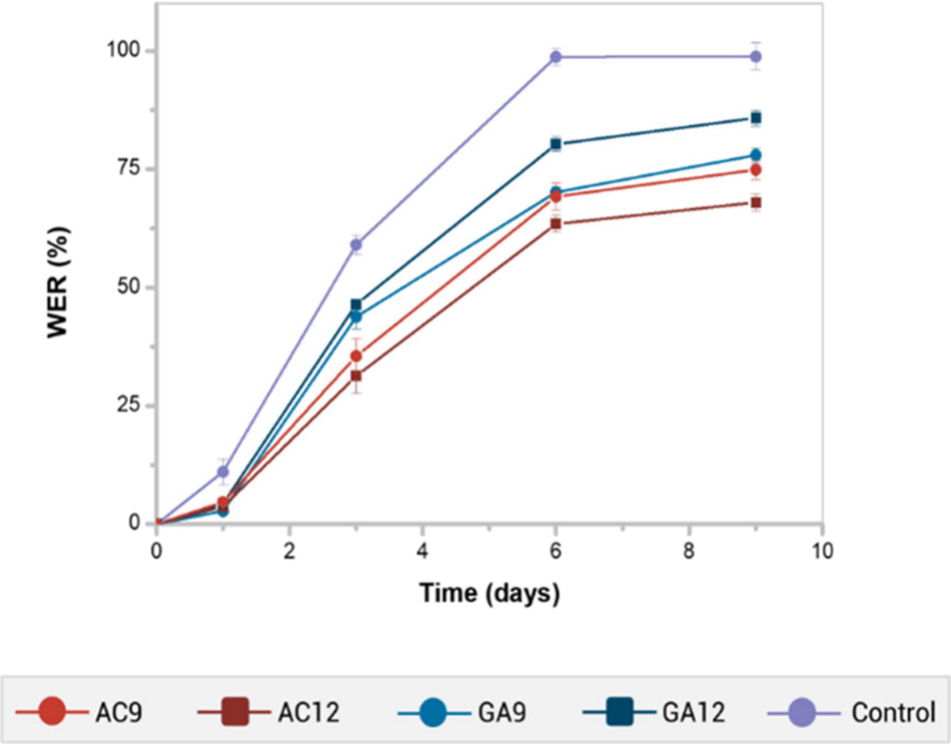
Water evaporation ratio of pure soil and soil containing the AC and GA hydrogels.

Figure 8 shows the WER values of hydrogel-added soils and the hydrogel-free soil over 9 days. The presence of 0.5 wt % of hydrogels enhanced the amount of water retained in all samples, but the AC hydrogels demonstrated a better capacity to preserve the humidity of soil. After 9 days, the WER values decreased in the following order: pure soil (98.80%), GA12-containing soil (85.90%), GA9-containing soil (78.00%), AC9-containing soil (74.90%), and AC12-containing soil (68.00%). This feature agrees with the swelling relative behavior of hydrogels; a higher swelling capacity of AC hydrogels (Figure 7b) induced a higher water retention of soil.

### Effects of Hydrogels on Plant Growth

The absence of phytotoxicity is the first requirement for using any material as a component of the growth medium of plants. Hydrogel samples with the highest HA content were used in the phytotoxicity test. The assays revealed that 83% of sorghum seeds germinated in the control sample and in the presence of GA12 hydrogel; meanwhile, 80% of the seeds germinated with the AC12 hydrogel. In commercial seeds, a value close to 90% is accepted.^56^ However, due to the seeds nature and the conditions of the assays, it is not expected that all seeds achieve germination. According to Montesano et al.,^6^ the lower limit of germination to consider a polymeric material as nonphytotoxic is 60%. From this point of view, the use of both GA12 and AC12 hydrogels is safe for plants. PVA-based hydrogels have been evaluated for agricultural applications, mixed with soil or for seed coating, evidencing a high water retention over long periods and a positive impact on the survival rate of plants.^52^ Lopez-Velazquez et al.^54^ have also reported that PVA hydrogels act either as water containers or as protective means for plants.

**Figure 9:**
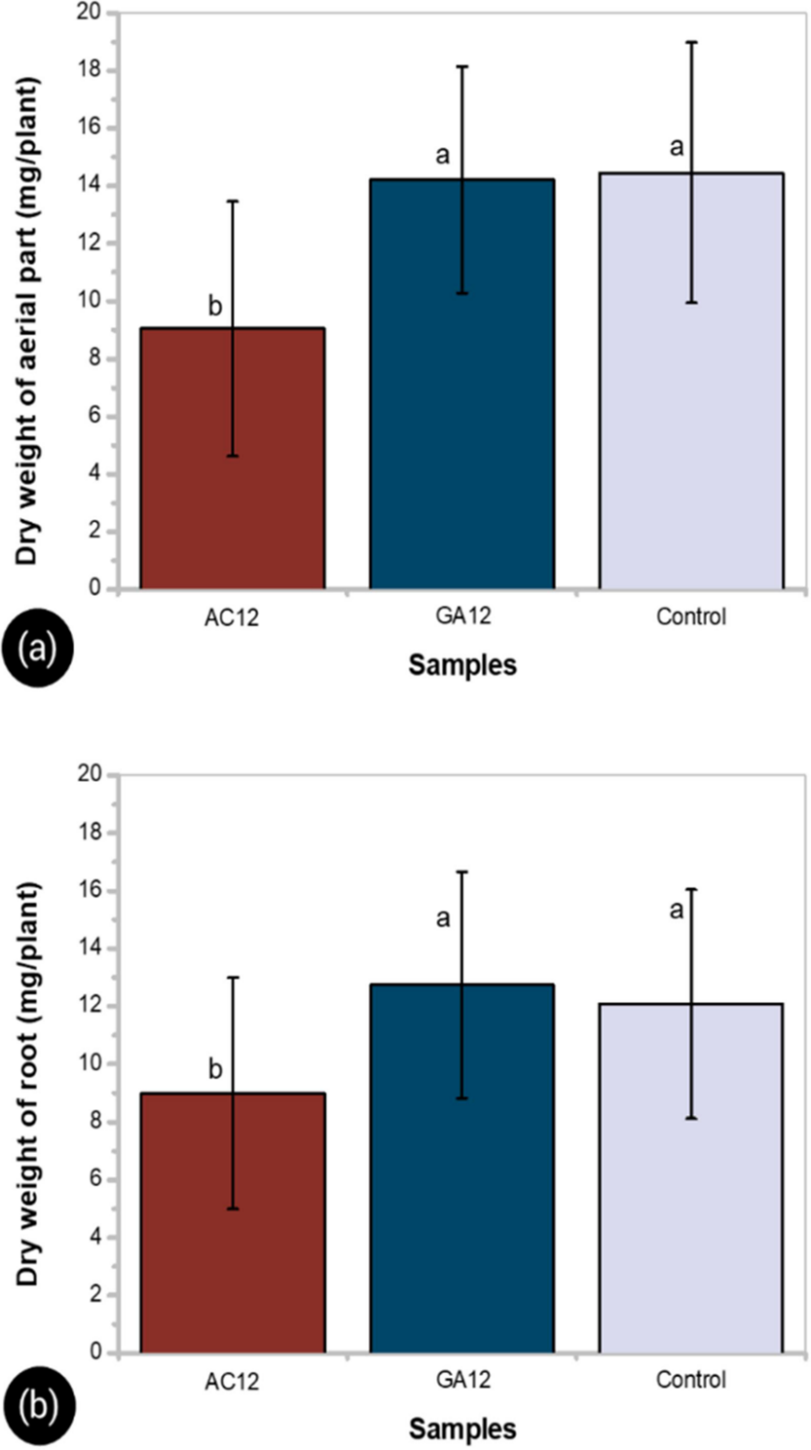
Effect of composite hydrogels on dry weight of root (a) and aerial part (b) of sorghum (*Sorghum sp.*). Different letters in the columns indicate significant differences at *P* < 0.05.

Figure 9 shows the average dry weight of the roots and aerial parts of sorghum plants that grew in the presence of GA12 and AC12 hydrogels. Statistical analysis showed significant differences in the dry weight of roots and aerial parts of sorghum according to the method used to prepare the PVA/HA hydrogels. The plant growth was promoted in the presence of the GA12 hydrogel as compared to the effect observed with the AC12 sample. No significant differences were detected in the dry weight of roots and aerial parts between the GA12 hydrogel and the control sample. The weight parameters were significantly lower when the AC12 hydrogel was used as compared to the control condition. It is expected that the microstructure of hydrogels improves the water availability and airflow through the system, reducing the drought stress and aeration problems for plants, respectively.^11^ For PVA/HA hydrogels, the heterogeneous porous structure of the PVA network formed by adding GA seems to promote the growth and storage of seedlings, as compared to the behavior of autoclaved hydrogels with lower pore dimensions and a highly uniform pore distribution.

### Urea Release

Most of traditional agrochemicals including fertilizers fail to reach the target site due to adverse processes such as leaching, volatilization, and air-drift.^27,57^ For instance, the nitrogen (N) use efficiency is around 40% for conventional urea.^58^ Significant N losses cause economic and environmental problems such as N_2_O emissions. In Mexican agriculture, N_2_O from cultivated soils represents one of the largest sources of emissions, eutrophication, and loss of soil fertility.^57^ Therefore, the use of urea-controlled release systems can be a potential solution to the problem of urea loss in agricultural crops.^27,35,59^

**Figure 10:**
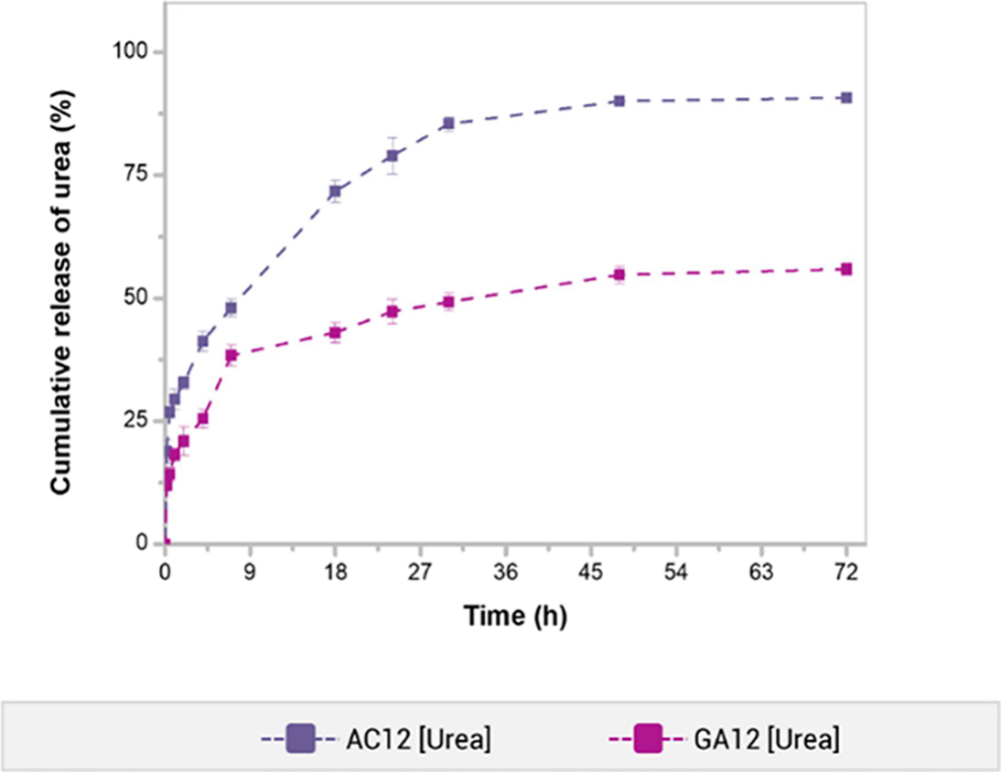
Urea release kinetics of AC12 and GA12 hydrogels; the experiments were performed in soil extract media at 25 °C.

Figure 10 shows the urea release kinetics from AC12 and GA12 hydrogels in a soil aqueous extract at room temperature. The preparation method of the PVA/HA hydrogel influenced the urea release behavior. The urea release profiles revealed an initial burst delivery up to the first 2 h, reaching 33% of urea cumulative release for the AC12 sample and a lower value of 21% for the GA12 hydrogel. It is notorious that the amount of urea released at equilibrium varied with respect to the sample, indicating a correlation with the preparation method. The AC12 hydrogel released 91% of the urea content within the first 72 h, while the amount achieved by the GA12 hydrogel was 56%. Both hydrogels exhibited a sustained release of urea up to 3 days. The release kinetic of urea and the total amount of the fertilizer at equilibrium were consistent with the swelling relative behavior of hydrogels. The AC12 hydrogel showed a more efficient urea release as compared to the GA12 hydrogel. This feature can be attributed to the flexibility of PVA chains in the hydrogels obtained by the autoclave process, in contrast to the chain mobility restrictions of the GA-cross-linked hydrogels.^7^

### Kinetic Model

To further examine the release mechanism of urea from the hydrogels, the experimental data obtained in the release studies were fitted to the model developed by Korsmeyer–Peppas. This model has been applied to evaluate the mechanisms involved in the chemical release process, as well as the possible coupling between the relaxation swelling of polymer systems and the diffusion phenomena of the model compound through the polymer matrix.^14,40^ Fitting parameters are summarized in Table 5.

**Table 5:** Fitting Parameters of Urea Release Data to the Korsmeyer–Peppas Equation

hydrogel sample	fitting parameters			release mechanism
	*n*	*k* (10^–3^)	*r*^2^	
AC12	0.24	7.99	0.983	Fickian diffusion
GA12	0.29	4.92	0.942	Fickian diffusion

The values of *n* indicated that the transport of urea through the hydrogels was controlled by a Fickian diffusion mechanism in both hydrogels, in agreement with the swelling mechanism of samples in aqueous media. It is also observed that the *k* value was 62% higher for the AC12 sample than for the GA12 hydrogel, evidencing the possibility of modulating the urea release kinetic from PVA/HA hydrogels by choosing between the preparation method of AC or GA cross-linking.

## Conclusions

PVA hydrogels were successfully synthesized by AC or by adding GA in the presence of different proportions of HA. FTIR confirmed the formation of a 3D-supramolecular network of PVA by the autoclaving/freezing process, as well as the presence of acetal bridges in the GA-mediated cross-linked hydrogels. The thermal stability of the AC hydrogels was affected by the autoclaving process, whereas the thermal behavior of the GA samples was improved due to the chemical cross-linking. All hydrogels showed an interconnected porous structure, though GA samples displayed a less homogeneous morphology than the AC samples. PVA/HA hydrogels increased the water content of soils; a higher swelling capacity of AC hydrogels induced a higher water retention of the soil. Both AC and GA hydrogels showed nonphytotoxicity during the growth test of *Sorghum sp*.; however, the plant growth was promoted in the presence of the GA hydrogel as compared to the effect observed with the AC sample, evidencing the key role of the hydrogel morphology in the agricultural application. PVA/HA hydrogels showed a sustained delivery of urea at 25 °C in soil extract by a classical diffusion mechanism, although the total amount delivered of the fertilizer was closely influenced by the hydrogel structure. Besides the compositional concerns, this work demonstrated that the preparation method is an important criterion in the design of hydrogels intended for the agricultural field. AC or GA-cross-linked PVA/HA hydrogels showed suitable characteristics for agricultural applications, and particular requirements of crops can be addressed by choosing the appropriate method for the preparation of material.

## Supporting Information Available

Values considered for network parameters of PVA hydrogels, photographs of compression–decompression test, and graphics of pore size distribution (PDF)
