# Supplementary material for: Physically and Chemically Cross-Linked Poly(vinyl alcohol)/Humic Acid Hydrogels for Agricultural Applications
Source: ACS Omega. 2023 Nov 15;8(47):44784–95. doi: 10.1021/acsomega.3c05868 (PMC10688162; doi:10.1021/acsomega.3c05868)
Supplement: Supplementary file 1 — ao3c05868_si_001.pdf [file ao3c05868_si_001.pdf]

Supporting Information

Physically and Chemically Cross-Linked Poly(vinyl alcohol)/Humic Acid Hydrogels for Agricultural Applications

Ana V. Torres-Figueroa[1], Sergio de los Santos-Villalobos[2], Dora E. Rodríguez-Félix[1], Sergio F. Moreno-Salazar[3], Cinthia J. Pérez-Martínez[4], Lerma H. Chan-Chan[5], Andrés Ochoa-Meza[3*], Teresa del Castillo-Castro[1*]*

[1]Departamento de Investigación en Polímeros y Materiales, Universidad de Sonora, Hermosillo 83000, Mexico.

[2]Laboratorio de Biotecnología del Recurso Microbiano, Departamento de Ciencias Agronómicas y Veterinarias, Instituto Tecnológico de Sonora, 5 de Febrero 818 Sur, Colonia Centro, Obregón 85000, Mexico.

[3]Departamento de Agricultura y Ganadería, Universidad de Sonora, Carr. Bahía de Kino, Km. 21. Apartado Postal 305, Hermosillo, Sonora, Mexico.

[4]Departamento de Ciencias Químico Biológicas, Universidad de Sonora, Hermosillo 83000, Mexico.

[5]Departamento de Física, CONAHCyT, Universidad de Sonora, Hermosillo 83000, Mexico.

*Corresponding authors email: anavaleria.torresf@gmail.com, andres.ochoa@unison.mx, teresa.delcastillo@unison.mx

Table S1. Values considered for network parameters of PVA hydrogels.

Parameter	Symbol	Value	Unit	Reference
Number average molecular weight of the dry polymer	$\bar{M}_n$	104,500	g/mol	a
Density of the dry polymer	ρp	1.27	g/mL	[1,2]
Molar volume of the solvent (water)	V_1	18	mL/mol	[1,2]
Flory's polymer-solvent interaction parameter	χ	0.494	N/A	[2]
Junction functionality	f	4	N/A	[2]
Ideal gas constant	R	8.314	J/K·mol	[3]
Temperature	T	298.15	K	b
Weighted average bond length	$\bar{l}$	0.154	nm	[2]
Flory characteristic ratio	C∞	8.3	N/A	[2,4]
Molecular weight of the repeating unit	$\bar{M}_r$	44	g/mol	[2,5]
Number of atoms in the repeating unit backbone	λ	2	N/A	c

a) Determined based on average of molecular weight of the reagent.
b) Determined based on ambient temperature.
c) Determined based on the repeating unit's chemical structure.

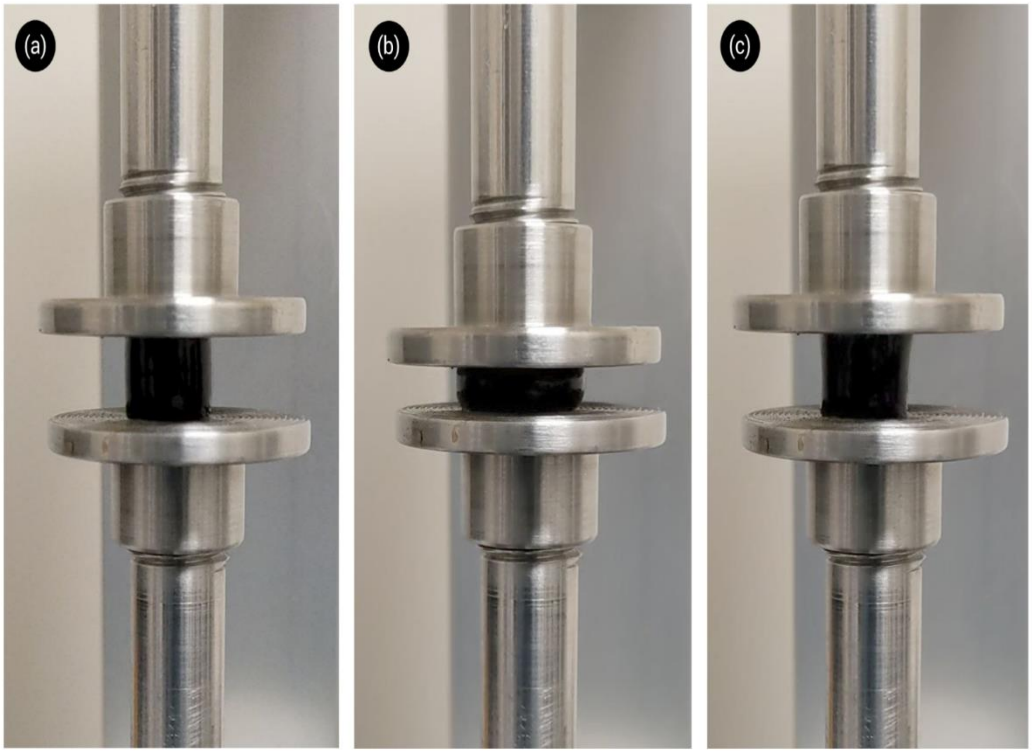

Figure S1. Photographs of the shape recovery of the hydrogels before the test (a), at 50% of deformation (b) and after the test (c).

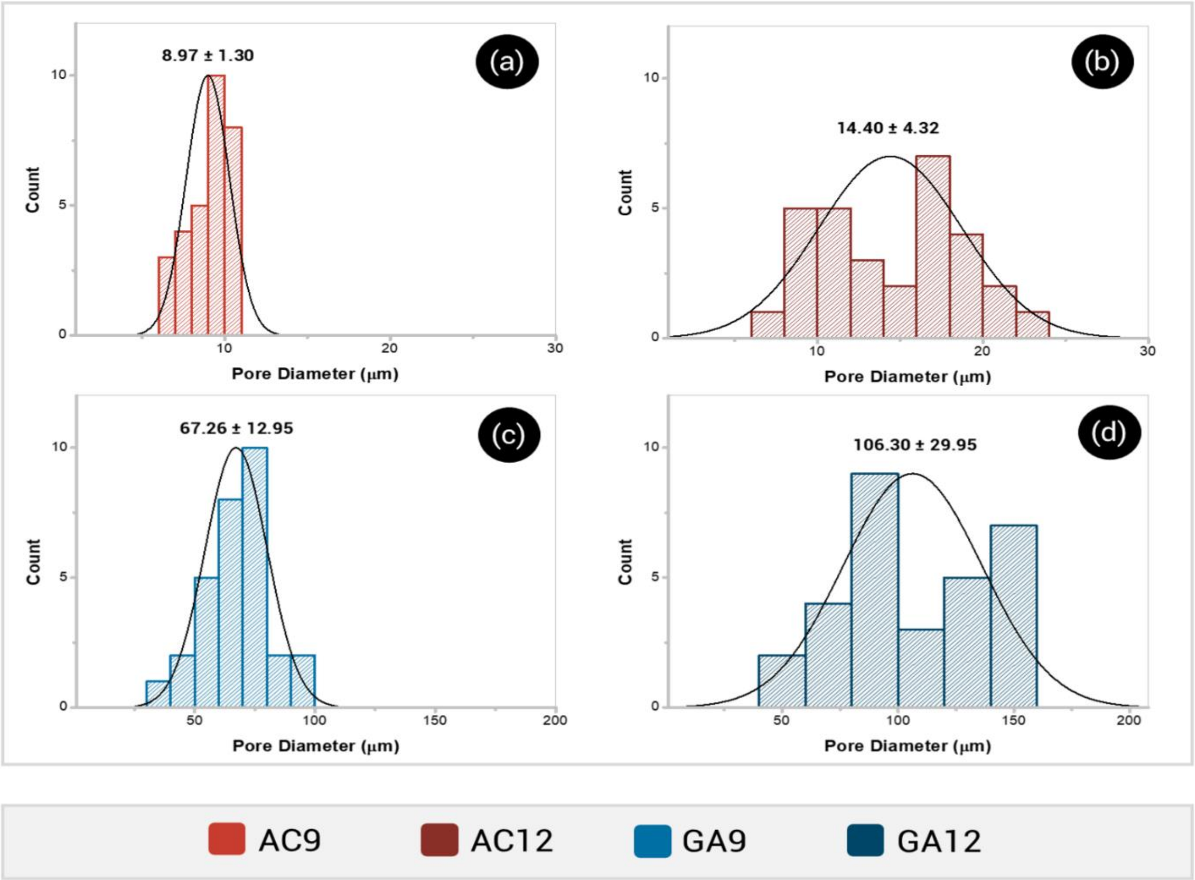

Figure S2. Pore size distribution of AC9 (a), AC12 (b), GA9 (c), and GA12 (d).

REFERENCES

(1) Canal, T.; Peppas, N. A. Correlation between Mesh Size and Equilibrium Degree of Swelling of Polymeric Networks. *J. Biomed. Mater. Res.* **1989**, *23* (10), 1183–1193. https://doi.org/https://doi.org/10.1002/jbm.820231007.

(2) Richbourg, N. R.; Wancura, M.; Gilchrist, A. E.; Toubbeh, S.; Harley, B. A. C.; Cosgriff-Hernandez, E.; Peppas, N. A. Precise Control of Synthetic Hydrogel Network Structure via Linear, Independent Synthesis-Swelling Relationships. *Sci. Adv.* **2021**, *7* (7). https://doi.org/10.1126/sciadv.abe3245.

(3) Pitre, L.; Sparasci, F.; Risegari, L.; Guianvarc'h, C.; Martin, C.; Himbert, M. E.; Plimmer, M. D.; Allard, A.; Marty, B.; Albo, P. A. G.; Gao, B.; Moldover, M. R.; Mehl, J. B. New Measurement of the Boltzmann Constant k by Acoustic Thermometry of Helium-4 Gas. *Metrologia* **2017**, *54* (6), 856. https://doi.org/10.1088/1681-7575/aa7bf5.

(4) Hickey, A. S.; Peppas, N. A. Mesh Size and Diffusive Characteristics of Semicrystalline Poly(Vinyl Alcohol) Membranes Prepared by Freezing/Thawing Techniques. *J. Memb. Sci.* **1995**, *107* (3), 229–237. https://doi.org/https://doi.org/10.1016/0376-7388(95)00119-0.

(5) Peppas, N. A.; Wright, S. L. Drug Diffusion and Binding in Ionizable Interpenetrating Networks from Poly(Vinyl Alcohol) and Poly(Acrylic Acid). *Eur. J. Pharm. Biopharm.* **1998**, *46* (1), 15–29. https://doi.org/10.1016/S0939-6411(97)00113-6.
